# SEC Based Method for Size Determination of Immune Complexes of Therapeutic Antibodies in Animal Matrix

**DOI:** 10.1155/2016/9096059

**Published:** 2016-07-31

**Authors:** Marta Boysen, Laura Schlicksupp, Ingeborg Dreher, Ralf Loebbert, Mario Richter

**Affiliations:** AbbVie Deutschland GmbH & Co. KG, Knollstrasse 50, 67061 Ludwigshafen, Germany

## Abstract

Therapeutic monoclonal antibodies (mAbs) represent a milestone in pharmacological development. Their superiority is based on the combination of high specificity, low toxicity, and long half-life that characterizes biologics. If biologics have Achilles' heel, it is their potential immunogenicity. To better understand the impact of the size of immune complexes of mAbs on anti-drug antibody (ADA) dependent adverse reactions in* Macaca fascicularis*, we developed an efficient high-throughput size exclusion chromatography- (SEC-) based methodology that enables analysis of the size, size distribution, and ratio of free and ADA-complexed mAb in serum allowing for assessment of formation and clearance of circulating ADA-mAb immune complexes (CIC).

## 1. Introduction

Therapeutic mAbs represent a mature medicinal technology with proven therapeutic benefit in many clinical disease indications. Their advantages over conventional small molecule drugs include their high specificity, long half-life, and low toxicity; however biologics are more likely than small molecule therapeutics to induce immune reactions. Fully humanized therapeutic mAbs, engineering advances resulting in fewer immunogenic CDR regions, improved formulations, and quality control and other developments have made therapeutic mAbs safer and highly beneficial for many patients around the world. Although many therapeutic mAbs still elicit an immune response in a certain subpopulation of patients (e.g., [1, 2]) and it is sometimes associated with hypersensitivity reactions (e.g., [3–7]), the clinical application of mAbs in general is well tolerated. However mAb-neutralizing immune response is not uncommon [8–10]. Therapeutic mAbs are tested for their safety (among other species) in nonhuman primates such as* Macaca fascicularis* (cynomolgus monkey) and have been observed to cause adverse reactions mediated by circulating immune complexes (CIC) formed by anti-drug antibody complexation with mAbs [11]. The immunogenicity and the associated risks of therapeutic antibodies in preclinical species are poorly predictive of their immunogenicity and risks in humans. One reason is the differences between the immune systems of humans and nonhuman primates [12]. Further, preclinical species may recognize the humanized backbone of therapeutic antibodies as foreign [13, 14]. Nevertheless, further characterization of CIC-dependent adverse reactions in preclinical species is of high interest to the pharmaceutical industry [15]. A more profound understanding of the underlying mechanisms would help to minimize the risks in animal models and the likelihood of a negative study outcome. One sequel of CIC-dependent postdose reaction (PDR) is activation of the complement system followed by inflammation, deposition of immune complexes, and subsequent tissue damage. CIC-mediated reactions are difficult to predict as many individual factors which differ among species and individuals (e.g., the ability to process, metabolize, and distribute CIC) contribute to the outcome. One factor, which might serve as a marker for CIC-dependent reactions, is the size of immune complexes [8, 16]. It has been shown that patients who develop an immune response against therapeutic antibodies without any adverse symptoms have small immune complexes, while patients with autoimmune diseases like lupus erythematosus have large immune complexes associated with aggressive disease progression [8, 17]. Various factors contribute to the size of immune complexes, including mAb concentration, ADA concentration, antigen concentration, the affinities of the complex components, oligomer status, number of binding sites of complex components, and clearance mechanisms [18].

**Figure 1:**
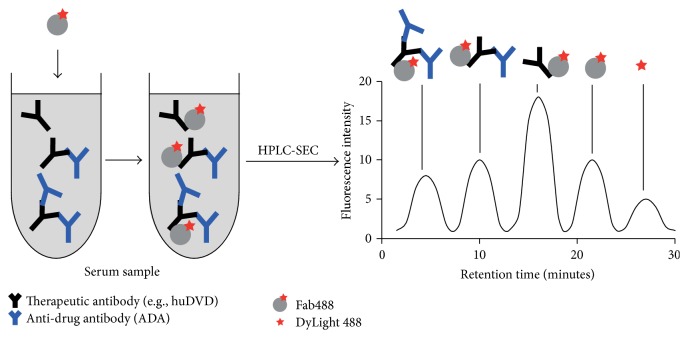
Principle of SEC assay. Fab488 is added to a serum sample. After incubation, Fab488 binds to the therapeutic mAb in the serum samples and to ADA complexes containing the therapeutic mAb (left). Formed complexes are separated by size exclusion chromatography (SEC) and the peaks quantified (right).

We developed an assay which characterizes the size, size distribution, and abundance of CIC in animal serum. The therapeutic mAb construct evaluated in these experiments was a humanized dual-variable domain immunoglobulin directed against soluble epitopes (huDVD) [19]. The assay was developed to assess CIC produced by cynomolgus monkeys following repeated parenteral administration of huDVD, some of which culminated in acute postdose hypersensitivity reactions. This assay enables quantification of free and complexed huDVD in one experiment. We used a DyLight 488-labeled Fab fragment of a monoclonal anti-human IgG_1_ antibody (Fab488) to detect huDVD in serum. To quantify and characterize free and complexed huDVD we used size exclusion chromatography equipped with a laser-induced fluorescence (LIF) detector (Figure 1). The method achieved a sensitivity in the low microgram per milliliter range and was suitable for monitoring enrichment and clearance of free huDVD and huDVD-containing complexes of various sizes. We also monitored total amount of huDVD by western blot and quantified free concentration using a ligand biding assay.

## 2. Materials and Methods

### 2.1. Generation of Fab Fragments

Purified anti-human IgG antibody (8 mL, 3.5 mg/mL, AbbVie proprietary monoclonal mouse antibody) in digestion buffer (Thermo Fisher Scientific) was incubated with immobilized papain (2 mL, Thermo Fisher Scientific, 20341) for 24 hours at 37°C. The papain beads were removed by centrifugation. The Fc fragment was removed using a 5 mL Protein A column (HiTrap rProtein A FF, 5 mL, 17-5079-01, GE Healthcare) in PBS as binding buffer.

### 2.2. Labeling of Fab Fragments with DyLight 488 for the Generation of Fab488

The purified Fab fragment (3 mg/mL) was labeled with a 10-fold molar access of DyLight 488 (Thermo Fisher Scientific, 20341) for 2 hours at room temperature in the dark. Excess label was removed on S200 16/600 column (28-9893-35, GE Healthcare) in PBS using ÄKTA-Explorer FPLC (GE Healthcare).

### 2.3. Purification of Polyclonal Antibodies Directed against the CDR of huDVD

Rabbits were immunized with huDVD (Eurogentec, 4 week immunization, “Speedy”) and the collected serum depleted of antibodies recognizing common human IgG epitopes in a two-step procedure. 100 mg of human IgG (Sigma Aldrich, I4506) was added to the serum (200 mL) together with a 5% w/v solution of polyethylene glycol 6.000 (Fluka, 81253) and incubated at 4°C for 12 hours. The serum supernatant was harvested by centrifugation (1500 ×g/4°C, 20 minutes) and filtration (0.45 *μ*m bottle-top filter, Merck Millipore) and incubated with 100 mg human IgG coupled to 4 mg CnBr Sepharose (GE Healthcare, 17-0430-01, coupling was performed according to the manufacturer's instructions (71-7086-00 AF)) for 2 hours at room temperature. The beads were removed using an Econo-Pac column (Bio-Rad Laboratories). The serum flow-through was incubated at 4°C for 12 hours with 100 mg of the drug antibody immobilized on 4 mg CnBr Sepharose. The beads were washed with 5 bed volumes of PBS and the bound polyclonal response was eluted with IgG elution buffer (Thermo Scientific, 21009). The elution was neutralized with 10% 2 M Tris pH 7.5. The purified polyclonal response was tested for drug specificity using an ELISA test.

### 2.4. Sample Preparation and Liquid Chromatography

An Alliance Waters 2795 system equipped with Bio-SEC-5 column (500 Å, 5190-2533, Agilent Technologies, Inc.) was used for liquid chromatography. All runs were performed at 0.2 or 0.3 mL/min in PBS. Serum samples were diluted 1 : 5 in a volume of 100–150 *μ*L. 585 or 678 nM Fab488 was used (in final volume).

### 2.5. Fitting Operations

The binding equilibrium data points were fitted to a one site specific binding equation *Y* = *F* + (*F*_max_ − *F*)*∗*((((*a* + *x* + *K*_*D*_)/2) − sqrt(((*a* + *x* + *K*_*D*_)/2)^2^ − *a∗x*))/*a*) using GraphPad Prism. *F*_max_ is the maximum value of *Y*, *F* is the minimum value of *Y*, *x* is the total concentration of huDVD, *a* is the total concentration of Fab488, and *K*_*D*_ is the equilibrium dissociation constant. The equation is the solution of the quadratic equation for [*AB*] derived from *K*_*D*_ = ([*A*]*∗*[*B*])/[*AB*], where [*A*] is the free concentration of Fab488, [*B*] is the free concentration of huDVD, and [*AB*] is the concentration of the complex of Fab488 bound to huDVD. The % bound huDVD was calculated using the specific binding equation and the best-fit value for *K*_*D*_.

### 2.6. Western Blot

Serum samples (0.5 *μ*L per well) were separated on Criterion TGX Stain-Free Precast gels (Bio-Rad Laboratories) at 250 V for 30 minutes. The proteins were transferred via Trans-Blot Turbo Transfer System (Bio-Rad Laboratories) onto a nitrocellulose membrane (Trans-Blot Turbo Transfer Pack, Bio-Rad Laboratories). The nitrocellulose membrane was blocked for 5 hours at room temperature in TTBS, 2% BSA, and incubated o/n at 4°C in TTBS, 2% BSA, 3% milk powder, and 50 ng/mL of the AbbVie proprietary monoclonal mouse anti-human IgG antibody. The membrane was washed 5 times for 5 minutes and 3 times for 10 minutes in TTBS and 0.1% BSA. The membrane was incubated for 1 hour at room temperature in the standard ultrasensitive ABC staining solution (Thermo Fisher). The nitrocellulose membrane was washed 5 times for 5 minutes, once for 10 minutes, and twice for 5 minutes in TBS buffer. The membrane was incubated for 3 minutes in the dark with SuperSignal West Femto Maximum Sensitivity Substrate (Thermo Fisher). The images were taken using VersaDoc Imaging System (Bio-Rad Laboratories). The analysis was done using Image Lab 5.1 software (Bio-Rad Laboratories).

### 2.7. SPR

All experiments were performed using a Biacore 3000 instrument (GE Healthcare) and buffer HBS-EP (GE Healthcare, BR100188). 1000 response units of anti-human IgG Fc antibody (Thermo Fisher Scientific, 31125) were immobilized on both flow cells of a CM5 chip (GE Healthcare, BR-1003-99) according to the manufacturer's instructions (GE Healthcare). After each injection cycle of huDVD over one flow cell (15 nM, 10 *μ*L) and Fab488 over both flow cells (0.49–2000 nM, 50 *μ*L/min, association 5 minutes, and dissociation 10 minutes) the chip was regenerated with 15 mM Glycine/HCl for 30 sec at 100 *μ*L/min.

### 2.8. Supernatant Pellet Experiment

678 nM Fab488, 830 nM huDVD, and 0, 1500, or 3000 nM ADA were incubated at 4°C for 5 h and 50 minutes to simulate the conditions of the SEC experiment for the sample, which was injected last. 30 *μ*L of each sample was centrifuged at 15000 rpm in a table top centrifuge for 10 minutes and 24 *μ*L were carefully removed. The samples were mixed with nonreducing sample buffer and subjected to SDS-PAGE. After Coomassie staining the bands were quantified using the Gel Doc Imager and the Image Lab 5.1 software (Bio-Rad Laboratories).

## 3. Results and Discussion

### 3.1. Generation of Fab488: A Fluorescence Labeled Anti-Human IgG Fab Fragment

**Figure 2:**
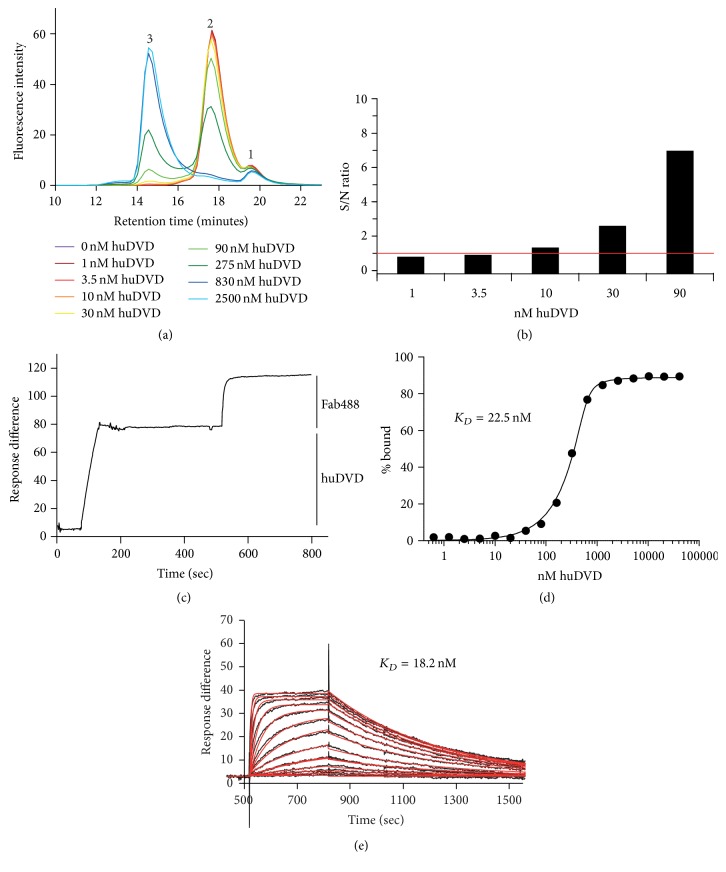
(a) Titration of huDVD on Fab488. 678 nM Fab488 and 1–2500 nM huDVD were incubated in 20% cynoserum as described in Section 2 and separated by size exclusion chromatography with fluorescence detection. Peak 1 is the free dye (DyLight 488), peak 2 is Fab488, and peak 3 is the complex of Fab488 and huDVD. (b) Determination of sensitivity. The signal to noise ratio (*S*/*N* ratio) of peak 3 was determined by dividing the percentage of the peak area of peak 3 in runs containing huDVD by the corresponding percentage of peak area at the same retention time of a control run containing no huDVD. (c) Stoichiometry. SPR signal showing the binding of huDVD to anti-human IgG Fc coated CM5 chip (0–200 sec) and the binding of Fab488 at saturation (500–800 sec). The average binding of huDVD was 78 response units and that of Fab488 37 response units (200000 and 47150 Da). (d) *K*_*D*_ of the binding of Fab488 to huDVD in 20% cynoserum determined by SEC. The % bound was calculated from the area of peak 3 as compared to total fluorescence as shown in experiment (a), plotted versus the concentration of huDVD, and fitted to the specific binding equation as described in Section 2 to determine the equilibrium dissociation constant (*K*_*D*_). (e) *K*_*D*_ of the binding of Fab488 to huDVD determined by SPR. Association (500–800 sec) and dissociation (800–1500 sec) of Fab488 (0.49–2000 nM) to and from huDVD bound to an anti-human IgG coated chip. The data points (black curve) were fitted to a 1 : 1 Langmuir binding model (red curve).

To be able to detect huDVD in the presence of animal serum containing high concentrations of endogenous immunoglobulins, we chose a mouse monoclonal in-house generated antibody that recognizes human IgG_1_ but not* Macaca fascicularis* IgG (data not shown). The mouse monoclonal antibody was digested with papain to generate monovalent Fab fragments that would not cross-link drug complexes and hence not create artificial complexes. The Fab fragment was purified and labeled with a tenfold molar excess of DyLight 488 and purified again to get rid of excess label to generate Fab488. The addition of Fab488 to untreated* Macaca fascicularis* serum resulted in the detection of 2 peaks: the main peak at 18 minutes (see Figure 2(a), peak 2), containing Fab488, and a smaller peak at 19.5 minutes, containing free DyLight 488 (see Figure 2(a), peak 1). The elution pattern of Fab488 corresponded well to its size (47150 kDa). Free DyLight 488 eluted later than expected for the calculated mass (1011 Dalton).

### 3.2. Characterization of the Interaction of Fab488 with huDVD Using SEC and SPR in 20% Naive* Macaca fascicularis* Serum

To determine the lower detection limit of huDVD by Fab488 we titrated huDVD from 0.23 to 500 *μ*g/mL (1–2500 nM), added 678 nM Fab488 and 20% untreated* Macaca fascicularis* serum pool to each sample, and analyzed complex formation using size exclusion chromatography (SEC) with fluorescence detection. At 678 nM Fab488 huDVD should be bound to almost 100% to Fab488 also at lower concentrations of huDVD, giving highest possible intensity of the complex peak. As expected, the peak of free Fab488 declined with higher huDVD concentrations (Figure 2(a), peak 2, 18 minutes), while the peak of the huDVD-Fab488 complex (Figure 2(a), peak 3, 15 minutes) increased with increasing concentration of huDVD, reaching a maximum between 830 and 2500 nM of huDVD when no free Fab488 was detected. Peak 1, the free dye, remained unaffected. The limit of detection was 2 *μ*g/mL of huDVD (10 nM) in the assay, which corresponded to 10 *μ*g/mL in 100% serum. At this concentration the *S*/*N* ratio of the complex peak was greater than 1.5 (Figure 2(b)).

To establish the stoichiometry of the interaction of Fab488 and huDVD, we immobilized about 80 response units of huDVD on an anti-human IgG Fc coated CM5 SPR chip, bound saturating levels of Fab488 to huDVD, and compared the relative amount of response units. For 1 : 2 (huDVD : Fab488) stoichiometry we expected 36 response units of Fab488 binding to 80 response units of huDVD. We measured 37 response units, which confirm the 1 : 2 binding model (Figure 2(c)).

To establish the equilibrium dissociation constant of Fab488 to huDVD in serum, we repeated the experiment form Figure 2(a) using 585 nM Fab488 and 0.32–20560.8 nM huDVD with 22 data points for better fitting results. Because of the sensitivity the concentration of Fab488 used cannot be lower than the estimated equilibrium dissociation constant (*K*_*D*_) (low nM range) and the lowest concentration of huDVD. However *K*_*D*_ can be fitted under these conditions using a one site specific quadratic binding equation derived from a 1 : 1 binding equilibrium, where the concentration of free huDVD is calculated as the total concentration minus the concentration of the complex. The concentration of huDVD was doubled for the fitting operation as compared to the concentration used in the experiment to account for the 1 : 2 stoichiometry based on the assumption that the binding of Fab488 to the 2 binding sites on one huDVD molecule is independent.  Figure 2(d) shows the percentage of the complex plotted versus the concentration series of huDVD and the fitted curve giving *K*_*D*_ of 22.5 nM.

To confirm the result we determined *K*_*D*_ with a kinetic experiment using SPR. 80 response units of huDVD were bound to an anti-human IgG Fc coated CM5 SPR chip and the association and dissociation of a titration series of Fab488 was monitored (Figure 2(e)). *K*_*D*_ was fitted using a global 1 : 1 Langmuir model and gave a value of 18.2 nM confirming the result from the SEC experiment.

### 3.3. SEC Analysis of Complex Sizes and Size Distribution of Purified Rabbit ADA Directed against huDVD and huDVD in the Presence of 20% Naive* Macaca fascicularis* Serum

**Figure 3:**
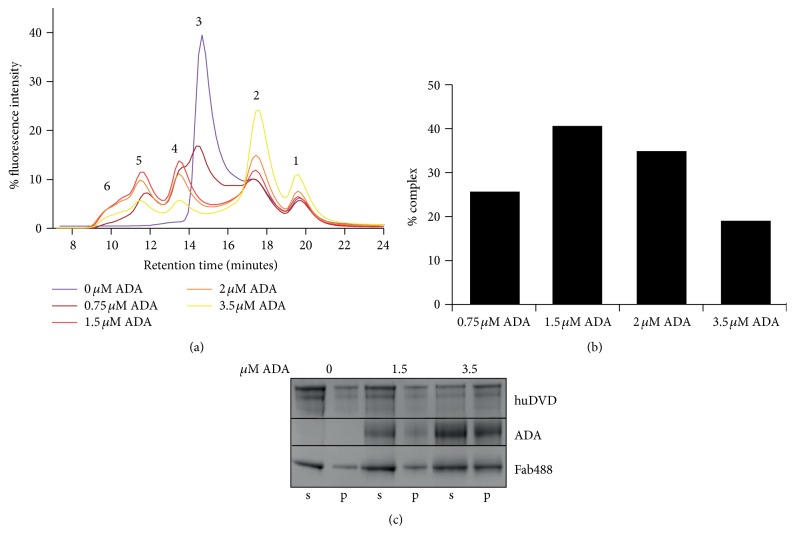
(a) Fab488-huDVD-ADA complexes prepared with ADAs purified from rabbit serum. 0.83 *μ*M huDVD, 678 nM Fab488, and 0.75, 1.5, 2, and 3.5 *μ*M ADA were incubated in 20% cynoserum as described in Section 2 and separated by size exclusion chromatography with fluorescence detection. Peak 1 is the free dye (DyLight 488), peak 2 is Fab488, peak 3 is the complex of Fab488 and huDVD, peak 4 is the complex of Fab488 and huDVD and one ADA, peak 5 is the complex of Fab488, one ADA, and two huDVD, and peaks under number 6 represent bigger complexes containing Fab488, huDVD, and ADA. (b) Quantification of fluorescence signal recovery in ADA complexes. Summed peak areas from (a) containing ADA (peaks 4–6) were set in relation to the fluorescence intensity of the whole elution profile and are shown as % complex. (c) Supernatant pellet experiment with ADA complexes. Coomassie stained SDS-PAGE showing the pellet (p) and the supernatant fraction (s) of samples containing 0, 1.5, or 3.5 *μ*M ADA, Fab488, and huDVD prepared as in (a). The samples were treated as described in Section 2. The pellet fraction contained 20% of the reaction volume, whereas the supernatant fraction contained 80% of the reaction volume. Aggregation occurs when more than 20% of total protein amount are in the pellet fraction.

To determine the resolution of free huDVD and different complexes of huDVD and ADA, we preincubated a constant concentration of huDVD (830 nM) with increasing concentrations of rabbit ADA. The rabbit ADA material contained only the fraction that recognizes the CDR regions of huDVD. The samples were subsequently incubated with Fab488 and separated by size exclusion chromatography as described in the previous experiment. When no ADA was added (Figure 3(a), dark purple trace) the only peaks detected were for free DyLight 488 (Figure 3(a), peak 1), free Fab488 (Figure 3(a), peak 2), and Fab488-huDVD complex (Figure 3(a), peak 3). Two additional peaks occurred at a substoichiometric ADA concentration (Figure 3(a), peaks 4 and 5, dark red trace). We interpreted these peaks as one huDVD bound to one ADA and two huDVDs bound to one ADA. With higher ADA concentrations, additional peaks at smaller retention times were detected (Figure 3(a), peak 6). These peaks could not be resolved any further with the chromatography setup employed. They increased with higher ADA concentrations (Figure 3(a), red, orange, and yellow trace).

At 3.5 *μ*M ADA we observed a relative increase in large complexes (Figure 3(a), yellow trace, peak 6); however we also observed an increase in free Fab488 (Figure 3(a), yellow trace, peak 2).  Figure 3(b) shows the relative amount of detected ADA-huDVD complexes in Figure 3(a). The amount of detected complexes of huDVD and ADA reached a maximum at 1.5 *μ*M ADA and was found to decline by up to 50% with higher ADA concentrations. To test whether the complexes precipitate with increasing ADA concentrations we repeated the experiment from Figure 3(a), fractionated the samples into a supernatant and pellet fraction, and analyzed the amount of proteins on SDS-PAGE (Figure 3(c)). Figure 3(c) shows that the amount of huDVD, ADA, and Fab488 increases in the pellet fraction (p) and decreases in the supernatant fraction (s). At the highest ADA concentration the quantification of the relative amounts of the proteins in the supernatant and pellet fractions showed that, at 3.5 *μ*M ADA about 40% of huDVD, about 20% of ADA and about 20% of Fab488 go to the pellet. This is close to the decrease in complex detection observed in the SEC experiment, showing that complex precipitation might be the main limitation in the analysis of immune complexes by this method.

### 3.4. Application of the SEC Method to* Macaca fascicularis* Samples from a Toxicology Study with huDVD

**Figure 4:**
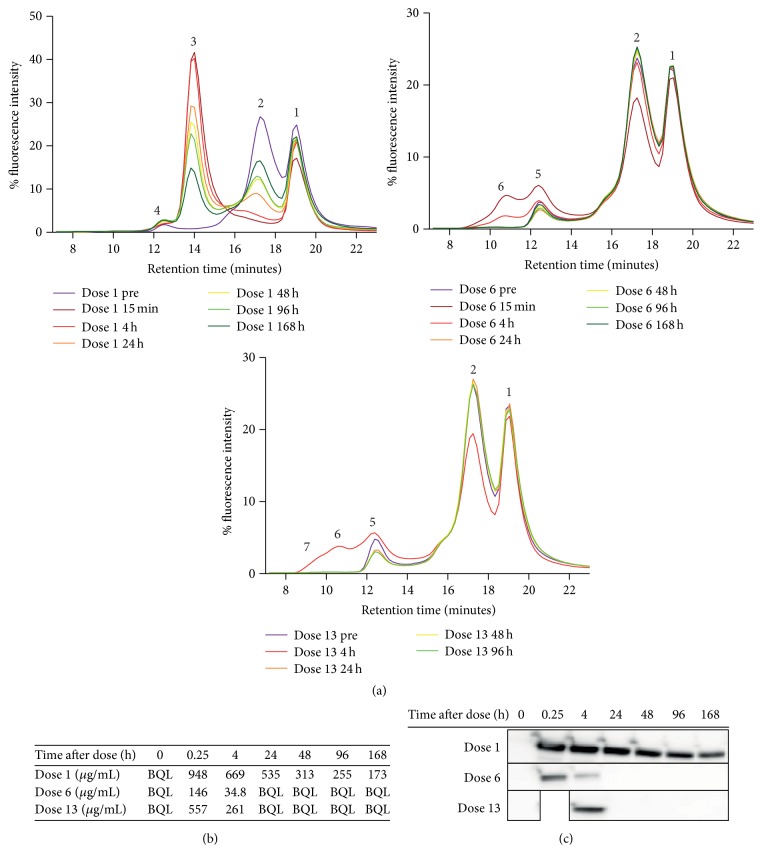
(a) SEC profile of toxicology study animal. 678 nM Fab488 was incubated with 20% serum samples from a cynotoxicology study with huDVD as described in Section 2 and separated by size exclusion chromatography with fluorescence detection. Peak 1 is the free dye (DyLight 488), peak 2 is Fab488, peak 3 is the complex of Fab488 and huDVD, peak 4 is unspecific binding, peak 5 is the complex of Fab488 and huDVD and one ADA, peak 6 is the complex of Fab488, huDVD, and two ADAs, and peaks under number 7 represent bigger complexes containing Fab488, huDVD, and ADA. Graph 1 shows the chromatographic traces of the predose sample (dose 1 pre) of one animal before the first dose and 15 minutes, 4 hours, 24 hours, 48 hours, 96 hours, and 168 hours after the first dose (from dark purple to dark green, dose 1 15 min–dose 1 168 h). Graph 2 shows chromatogram traces of the predose sample (dose 6 pre) of the same animal before dose 6 and 15 minutes, 4 hours, 24 hours, 48 hours, 96 hours, and 168 hours after the 6th dose (from dark purple to dark green, dose 6 15 min–dose 6 168 h). Graph 3 shows the chromatographic traces of the predose sample (dose 13 pre) of the same animal before dose 13 and 4 hours, 24 hours, 48 hours, and 96 hours after the 13th dose (from dark purple to green, dose 13 15 min–dose 13 96 h). The 15 minutes and 168 hours postdose samples were missing here. (b) PK profile of the same study animal measured with ligand binding assay. The concentration in *μ*g/mL of huDVD was measured with an antigen-based ligand binding assay using Mesoscale Discovery ECL technology. The figure depicts the concentration in 100% serum in the predose and postdose samples of doses 1, 6, and 13 for the same animal as in (a). BQL indicates that the values were below the lower limit of quantitation. (c) Relative quantitation of total amounts of huDVD with western blot. The same study samples as in (a) and (b) were separated by nonreducing SDS-PAGE and analyzed with western blot as described in Section 2. The huDVD signal is shown.

Having demonstrated that the method described above enables specific detection of free huDVD and ADA-huDVD complexes in spiked animal serum, we then applied the method to toxicology study samples. The animals received 20 mg/kg of huDVD weekly for a total of 13 doses, subcutaneously, as an intravenous slow infusion or as a bolus intravenous infusion, and then entered an eight-week dose free recovery period. All study samples were analyzed for free drug and free ADA levels using conventional ligand binding assays. At the 1st, 6th, and 13th dose, a predose sample and samples at 15 minutes, 4 hours, 24 hours, 48 hours, 96 hours, and 168 hours after dose were taken.  Figure 4(a) shows an example of an SEC profile. The animal in Figure 4(a) developed high ADA titers after dose 4. The first graph shows the chromatogram traces of the predose and postdose samples at dose 1. In the predose sample only the free dye peak (peak 1), the Fab488 peak (peak 2), and a small peak (peak 4) were detected. Peak 4 was present in all study animals and was likely a cross-reactivity of a serum component with Fab488. A Fab488-drug complex peak occurred in the following postdose samples (peak 3) which reached its highest intensity in the 15 minutes after dose sample (dark red chromatogram trace). The peak's intensity declined at later postdose time points.  Figure 4(a), second graph, shows the chromatogram traces of the monkey predose and postdose samples at dose 6. No peak of the Fab488-drug complex was detected here (peak 3 in the first graph), but additional broad peaks were detected in 15 minutes and 4 hours after dose samples (peaks 5 and 6), which disappeared again at later postdose time points. These peaks corresponded to peaks 4 and 5 from Figure 3(a); therefore the complexes likely contain 1 ADA and 2 ADA molecules.  Figure 4(a), third graph, shows the chromatogram traces of the monkey predose and postdose samples at dose 13. Similarly to dose 6, a specific huDVD-containing peak was only detected 4 hours after dosing. The peak pattern and abundance were very similar to the corresponding time point after dose 6, but there was also a significant broadening of the peak toward shorter retention times, indicating the existence of even larger immune complexes after dose 13. We had found that the binding of Fab488 to huDVD was in equilibrium after 1 hour and remained stable for at least 12 hours. Therefore we analyzed the predose and postdose samples of one animal from doses 1, 6, and 13 in one automated series, which took 10 hours and 50 minutes. We did not explore whether Fab488-huDVD and the fluorescence signal itself were stable for more than 12 hours.

### 3.5. Determination of Total Amount of huDVD with Western Blot and Free Concentration of huDVD with a Ligand Binding Assay and Comparison to the Results of the SEC Method

Next, we asked whether the decrease of the fluorescence signal of huDVD complex peaks beyond 4 hours after dose after doses 6 and 13 correlates with total huDVD clearance. The western blot in Figure 4(c) shows total amount of huDVD in the same study samples as analyzed in Figure 4(a). The relative clearance of total huDVD is very similar to the clearance of huDVD-Fab488 and huDVD-Fab488-ADA complexes from the SEC experiment in Figure 4(a).  Figure 4(b) shows huDVD concentrations measured by ligand binding assay. The ligand binding assay was a bridging assay using one antigen to capture and the second antigen for detection. The lower limit of quantitation of the assay was 136 ng/mL in 100% serum; however the majority of measured concentrations were in the range of micrograms per milliliter, because the level dropped fast below the lower limit of quantitation. In all the samples that had measurable concentrations of huDVD in the ligand binding assay, we were also able to measure huDVD with the SEC assay. This was consistent with the SEC assay sensitivity of 10 *μ*g/mL.

## 4. Conclusions

This paper demonstrates a method for fast and automated SEC based measurement of the concentrations of therapeutic mAbs and of therapeutic mAb complexes in the serum of animals. We present an engineered monovalent detection reagent that specifically detects humanized therapeutic mAbs with IgG_1_ backbone in animal serum and complexes thereof. We showed that complexes formed with purified rabbit ADA material elicit a peak pattern very similar to the huDVD complexes in real samples of a toxicology study. We resolved three distinct complex sizes most likely representing complexes containing one ADA, two ADAs, and more ADAs. Finally, we showed that the sensitivity of our method is sufficient to be easily applied to toxicology studies in* Macaca fascicularis* to quantify therapeutic mAbs and therapeutic mAb complexes and their size distribution over time. We demonstrated that the immune complexes of a therapeutic mAb at late dosing time points in* Macaca fascicularis* are very large compared with the complexes documented for patients [8].

The advantages of the SEC based method with drug specific fluorescence detection are manifold: sample preparation is simple, involving only a sample centrifugation step and the addition of Fab488. A much lower sample dilution is required than with classical ligand binding assays, with the result that binding equilibria are only minimally perturbed and closer to real* in vivo* conditions. Further free and complexed mAb can be detected and quantified simultaneously. In contrast to the conventional ADA ligand binding assays routinely applied in industry, this method only quantifies ADA-mAb complexes and not free ADA, which often persists in the circulation after the therapeutic mAb has been cleared. Since the complexes are considered the potential pathogenic species, this assay allows not only monitoring the critical species but also keeping track of clearance of free therapeutic mAb.

The detection antibody used in this study, the Fab fragment of an anti-human IgG antibody with no cross-reactivity to* Macaca fascicularis* IgG, offers the opportunity of an generic assay setup as it binds to all humanized IgG_1_ type therapeutic mAbs in any animal matrix. One cannot rule out the possibility that high ADA levels might impact the assay readout, because the accessibility of the binding site of the detection reagent on the mAb might be sterically hindered in large complexes. Additional use of Fab fragments against other constant epitopes and a reagent with very high affinity to partially outcompete bound ADA could improve complex detection. Nevertheless compromised detection of very large complexes can never be ruled out. Conventional ADA ligand binding assays also suffer from limitations, namely, limited drug tolerance, which can never be excluded completely although acid dissociation steps are employed. We found that larger ADA complexes formed using polyclonal rabbit ADAs tend to precipitate. The SEC method can only analyze complexes, which stay soluble. In addition we were not able to resolve large complexes containing more than three antibodies, although we could observe an increase in the large unresolved species formed* in vitro* with rabbit ADA or in* in vivo* in cynomolgus monkeys. Recent findings suggest that pathologic immune complexes might contain no more than 6 ADAs [18]. If our method could detect these species it is not clear however. Therefore the resolution of the method has still to be improved.

The sensitivity of our method was 10 *μ*g/mL in 100% serum, which turned out to be very well suitable for the toxicology study analyzed here. We found that the complex peak detected at 10 *μ*g/mL contains 98% of the total concentration of huDVD bound to Fab488. Hence, better sensitivity could only be achieved by enhancing the brightness of the Fab fragment and improving peak resolution and the signal to noise ratio. This could be achieved by lowering the concentration of Fab488, engineering a protein with more label conjugation sites, and using capillary columns.

In summary, we developed a new generic method for the detection of humanized therapeutic mAbs in animal matrix such as serum which allows the detection of free therapeutic mAbs and ADA-mAb complexes in one analytical run, thereby enabling the measurement of the clearance of both species. In addition this method has a high throughput with very low instrument and consumables costs.
